# Evaluation of 2D-/3D-Feet-Detection Methods for Semi-Autonomous Powered Wheelchair Navigation

**DOI:** 10.3390/jimaging7120255

**Published:** 2021-11-30

**Authors:** Cristian Vilar Giménez, Silvia Krug, Faisal Z. Qureshi, Mattias O’Nils

**Affiliations:** 1 Department of Electronics Design, Mid Sweden University, Holmgatan 10, 851 70 Sundsvall, Sweden; silvia.krug@imms.de (S.K.); mattias.onils@miun.se (M.O.); 2 System Design Department, IMMS Institut für Mikroelektronik- und Mechatronik-Systeme Gemeinnützige GmbH (IMMS GmbH), Ehrenbergstraße 27, 98693 Ilmenau, Germany; 3 Faculty of Science, University of Ontario Institute of Technology, 2000 Simcoe St. N., Oshawa, ON L1G OC5, Canada

**Keywords:** 3D object recognition, YOLO, YOLO-Tiny, 3DHOG, histogram of oriented gradients, ModelNet40, feature descriptor, Intel RealSense, depth camera, wheelchair

## Abstract

Powered wheelchairs have enhanced the mobility and quality of life of people with special needs. The next step in the development of powered wheelchairs is to incorporate sensors and electronic systems for new control applications and capabilities to improve their usability and the safety of their operation, such as obstacle avoidance or autonomous driving. However, autonomous powered wheelchairs require safe navigation in different environments and scenarios, making their development complex. In our research, we propose, instead, to develop contactless control for powered wheelchairs where the position of the caregiver is used as a control reference. Hence, we used a depth camera to recognize the caregiver and measure at the same time their relative distance from the powered wheelchair. In this paper, we compared two different approaches for real-time object recognition using a 3DHOG hand-crafted object descriptor based on a 3D extension of the histogram of oriented gradients (HOG) and a convolutional neural network based on YOLOv4-Tiny. To evaluate both approaches, we constructed Miun-Feet—a custom dataset of images of labeled caregiver’s feet in different scenarios, with backgrounds, objects, and lighting conditions. The experimental results showed that the YOLOv4-Tiny approach outperformed 3DHOG in all the analyzed cases. In addition, the results showed that the recognition accuracy was not improved using the depth channel, enabling the use of a monocular RGB camera only instead of a depth camera and reducing the computational cost and heat dissipation limitations. Hence, the paper proposes an additional method to compute the caregiver’s distance and angle from the Powered Wheelchair (PW) using only the RGB data. This work shows that it is feasible to use the location of the caregiver’s feet as a control signal for the control of a powered wheelchair and that it is possible to use a monocular RGB camera to compute their relative positions.

## 1. Introduction

Powered wheelchairs (PWs) have improved the quality of life of many disabled people by providing them more independence and greater transport means, reducing their dependence on caregivers. The next step in PWs’ development is to improve their usability and the safety of their operation by integrating new features such as obstacle avoidance or autonomous driving control. This involves the use of additional sensors and electronic systems to measure the PW’s environment, detect objects, and determine their relative positions. However, fully autonomous PWs have many challenges and navigation limitations due to the wide variability of indoor and outdoor scenarios, lighting conditions, and obstacles, which limit their applications in practice. Despite the technological limitations, image-processing techniques enable new applications to control PWs to improve their usability. This work is focused on the development of a semi-autonomous contactless control of PWs that uses the position of a caregiver as a reference control in a side-by-side configuration. A camera system detects, recognizes, and tracks the caregiver walking beside the PW by measuring the caregiver’s relative distance and position with respect to the PW. Contactless control improves the PW’s operation when the user cannot properly control the PW, as well as improves the communication between the caregiver and the PW user, and thus the user’s quality of life [1].

Computer vision has become a cutting-edge research topic in recent years, especially in applications of autonomous robot navigation. Since the popularization of consumer depth cameras, image-processing and object-recognition techniques have been extended to include 3D data in the recognition pipeline. According to the measurement requirements of the contactless steering control of PWs presented in previous research [2], depth cameras allow measuring relative distances while capturing visual images of the PW’s environment, which makes them a good candidate for our application. However, 3D computer-vision techniques can be very computationally expensive and, therefore, pose a severe limitation for real-time operation in embedded systems.

Currently, there are two different approaches for object recognition: (1) hand-crafted features and (2) deep learning. In previous research, we evaluated the performance and limitations of a hand-crafted approach to object recognition based on an extension of the 3D data of a histogram of oriented gradients (3DHOG) object descriptor in combination with a Support Vector Machine (SVM) classifier [3,4,5]. The results showed relatively good recognition performance and good processing times, making it suitable for real-time operation. However, hand-crafted approaches require additional image preprocessing to segment the objects from the 3D data, which can lead to misclassification depending on the performance.

In this paper, we compared the 3DHOG approach with a deep-learning approach based on YOLOv4-Tiny to detect the feet of a caregiver next to a PW as the base input for semi-autonomous PW navigation. To compare both approaches, we constructed Miun-Feet—a custom dataset of images of caregiver’s feet taken from the PW in different scenarios, with different backgrounds and lighting conditions. The scientific contributions of this paper are as follows: (1) the generation of the Miun-Feet dataset using a depth camera placed on the PW in real working conditions with synchronized depth, RGB, RGB+depth, and point cloud data frames, including labels for the caregiver’s feet; (2) a method to segment the caregiver’s feet from the point cloud data using the RGB labels; (3) the evaluation of YOLOv4-Tiny using the input depth, RGB, and RGB-depth images; (4) a comparison of the hand-crafted 3DHOG and deep-learning YOLOv4-Tiny approaches in different scenarios and lighting conditions; (5) the proposed alternative method to compute the caregiver’s distance and angle using the RGB data channels.

## 2. Related Works

There has been extensive research on autonomous control and navigation for PWs. The research has focused on developing control systems and human–machine interfaces to facilitate the PW’s control in the case of severe mobility impairments [6,7,8] and autonomous navigation in controlled environments [9,10,11]. Contactless control of PWs using the caregiver as a control reference was explored in [12,13] using a combination of three LiDAR sensors and an omnidirectional camera placed on a pole. The detection and position measurement of the caregiver were performed using the LiDAR sensors, which measured the human chest profile represented by an ellipse, while the omnidirectional camera distinguished the caregiver from the other people next to the PW. Despite the good detection performances, it was difficult to integrate all the required sensors and electronic systems into the wheelchair due to the limited installation space for an additional embedded system as well as the limited power supply. In addition, placing the camera on a pole modified the ergonomics and appearance of the PW, making it unsuitable as a commercial product. A better camera placement was shown in [14], where a stereo-camera was mounted on the armrest of the PW. However, a lower camera placement does not allow the measurement of the complete human body shape due to limitations of the camera’s field of view (FoV). Therefore, caregiver detection was performed by detecting the caregiver’s legs.

On the other hand, 3D object recognition has been a fundamental research topic for computer vision since the popularization of consumer depth cameras and 3D object databases [15,16]. Besides an RGB camera, depth cameras enable a better understanding of the environment and, thus, a variety of applications, such as autonomous robot navigation [17], aerial robot surveying [18], or 3D shape reconstruction [19].

Three-dimensional recognition methods are generally categorized into (1) hand-crafted approaches and (2) deep learning approaches. Hand-crafted approaches are the classical computer vision techniques for object recognition. They are based on a combination of an object descriptor and a machine learning classifier. Thus, an object descriptor first extracts a key set of features from the objects and later a classifier estimates the class to which the object belongs. Generally, hand-crafted descriptors are categorized as global or local descriptors. Local descriptors focus on the local neighborhood and geometry of the keypoints of interest. They require the detection of the keypoints that contain information about their class and encode the local geometric information around them with a multidimensional feature vector [20]. Some of the popular local descriptors for 3D object recognition are the SIFT [21], SGCs [22], SHOTS [23], or RCS [24]. Local descriptors are robust to occlusions and therefore suitable for 3D object recognition in cluttered scenes. However, they require detailed object resolution to identify the keypoints, and therefore, they are not suitable for depth cameras due to the lower image resolution of the depth channel compared to an RGB camera. In contrast, global descriptors encode the entire object data in a single feature vector rather than just key-points of the object. Therefore, they require a prior object segmentation pre-processing before their computation but are less computationally and memory demanding than local descriptors. An example of global descriptors for 3D object recognition are: SI [25], VFH [26], FPFH [27], ESF [28], VFD [29], TriLCI [30] or the 3DHOG [3]. Three-dimensional hand-crafted descriptors perform well when objects have detailed shape information, but they are unable to adapt to complex shapes and scenes, limiting their success for uncontrolled scenarios such as autonomous navigation [31].

Current research in 3D object recognition is turning to deep learning approaches due to their higher recognition accuracy compared to hand-crafted approaches. Deep learning approaches are based on convolution neural networks (CNNs) that directly extract a hierarchical set of abstract features to maximize recognition accuracy. Deep learning approaches are categorized depending on the representation the object data as: (1) Voxel-based methods, e.g., VoxNet [31], ShapeNets [16], VoxelNet [32], or (2) Point-set-based methods, e.g., PointNet [33], FoldingNet [34] and, finally, (3) View-based methods, e.g., RotationNet [35], HMVCM [36] and Complex-YOLO [37]. Voxel-based and Point-set-based methods, also referred to as model-based methods [38], use a 3D volumetric CNN, and therefore, they exploit the 3D geometry of the objects. However, volumetric CNNs have a large and complex CNN architecture and require high computational and memory resources, and these are therefore not suitable for real-time applications. View-based methods, instead, transform the 3D objects into a series of 2D images from different viewpoints. As a consequence, they use 2D CNN methods and do not fully exploit the 3D data geometry of the objects, although they achieve good recognition performances, especially in the case of occlusions [38]. An alternative method for object recognition by a depth camera is to include the depth channel along RGB channels (RGB-D) in combination with a 2D CNN [39] and recursive neural networks (RNNs) [40] or encode the depth channel in jet color maps and the surface of normals [41]. Consequently, RGB-D methods for 3D recognition do not fully exploit 3D geometric information, but they reduce the hardware requirements compared to model-based methods and thus enable real-time applications, which is the intended use for contactless PW control.

## 3. Methodology

### 3.1. Application Description

**Figure 1:**
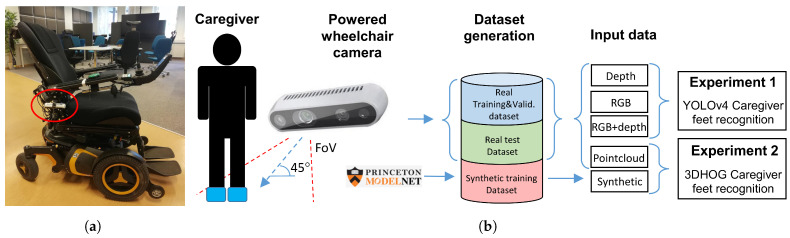
Power wheelchair setup and description of feet recognition approach. (**a**) PW with a depth camera below the armrest. (**b**) Caregiver feet recognition description.

The application description is shown in Figure 1b. The caregiver location is identified by detecting the position of the caregiver’s feet next to the PW. The required accuracy for the caregiver measurement is defined by the human social walking behavior [42,43], which is, therefore, between 0.7 and 1 meters. Feet recognition allows reducing the required FoV of the camera when detecting full size body shapes [44]. In addition, feet recognition enables the use of ground-plane (GP) detection and removal algorithms to segment the feet above the GP when using a hand-crafted recognition approach.

**Figure 2:**
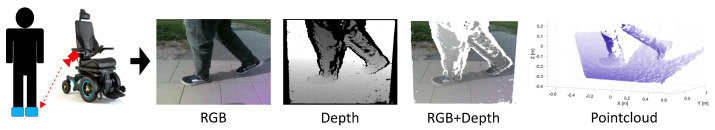
Camera placement on a wheelchair armrest and different camera outputs.

We chose the active depth camera Intel RealSense D455 [45] and installed it under the PW right armrest, tilted down 45 degrees with respect to the floor, as shown in Figure 1a and Figure 2. This placement allows for good measurement of the caregiver’s feet without compromising the ergonomics and appearance of the PW. To evaluate the influence of ambient lighting, we also placed a LED lighting system in the armrest of the PW, next to the depth camera, to illuminate the scene. The D455 depth camera provides aligned RGB and depth streams with global shutter for both the stereo and RGB cameras, enabling the application of object recognition in a moving environment [2]. In addition, camera software provides the necessary functions to compute the point cloud 3D representation of the aligned RGB and depth images. The configuration parameters of the D455 depth camera are listed in Table 1.

### 3.2. Experiments Definition

**Figure 3:**
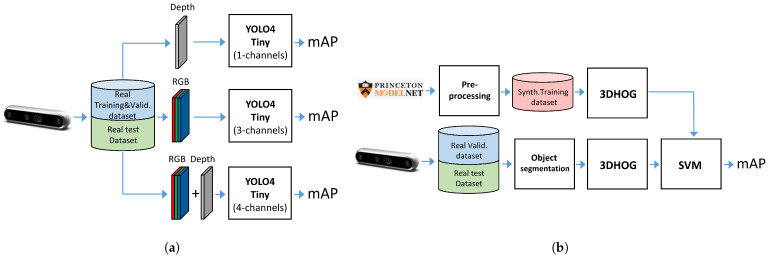
Experiment definition. (**a**) Experiment 1: YOLOv4-Tiny. (**b**) Experiment 2: 3DHOG.

We defined the following experiments in order to compare a hand-crafted approach as an object recognition approach with a deep learning approach:

- Experiment 1: We used the Miun-Feet custom dataset of images captured by a depth camera for training, validation and test in combination with a YOLOv4-Tiny-based approach for object recognition. We evaluated the different camera output formats in terms of Mean Average Precision (mAP). Therefore, as camera outputs, we use: (1) the depth channel (1 channel), (2) the visual RGB channels (3 channels), and finally, (3) a combination of RGB and depth (4 channels) to recognize the caregiver’s feet walking next to the PW, Figure 3a.
- Experiment 2: We used a synthetic dataset of objects segmented from the Modelnet40 dataset [16] for training and validation and the Miun-Feet dataset used in Experiment 1 for testing. We evaluated a hand-crafted approach based on the 3DHOG in terms of mAP. See Figure 3b for the voxel grid resolutions of $20^{3}$, $30^{3}$ and $40^{3}$ Voxels.
- Experiment 3: We propose an alternative method to measure the relative distance and angle of the caregiver beside a PW using the RGB camera output. We compared the results with respect to the ground truth data measured by the depth camera.

### 3.3. Miun-Feet Dataset Construction

**Figure 4:**
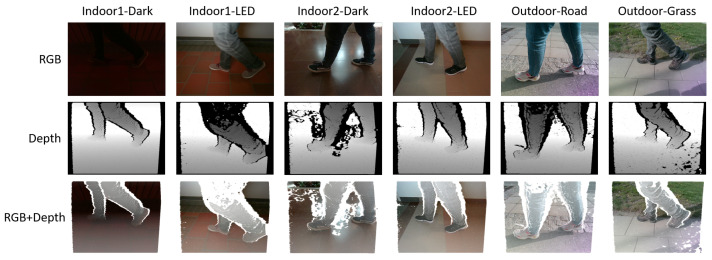
Miun-Feet dataset examples in different scenarios and shoes models.

The Miun-Feet custom dataset was generated by measuring a caregiver walking next to the PW using a depth camera Intel RealSense D455. The dataset contains images with different shoes, backgrounds and lighting conditions. Figure 4 provides examples for each configuration. The scenarios used for the data generation are summarized in Table 2. Additionally, we recorded empty frames (without feet) for training purposes. The captured data include ground truth distance labels that can be used during model training and evaluation.

For each camera scenario measurement, we collected the following data:

- RGB frames: Visual data frames (3 channels);
- Depth frames: Depth map image that includes true distances measurements from the camera for each pixel value (1 channel);
- RGB-D frames: 4-channels image that stacked visual frames (3 channels) and depth frames;
- Point cloud: Unstructured 3D data representation.

Based on the collected data, we construct sub-datasets for training, validation and testing by selecting the frames randomly and balancing the data according to the lighting conditions and eight different shoes models, as shown in Table 3 and Table 4. We include ground truth labels for both model training and validation datasets using LabelImg, also considering feet occlusions.

### 3.4. Synthetic Dataset Generation

**Figure 5:**
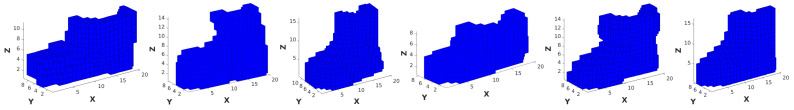
Examples of different synthetic objects segmented from the ModelNet40 dataset, including the frontal-view post-processing using a voxel grid of $20^{3}$ voxels.

To compare this work to the results obtained in a previous 3DHOG evaluation [4,5], we use a synthetic dataset for Experiment 2 (Figure 3b) to train the SVM classifier when using the 3DHOG object descriptor. We extracted the synthetic feet data from the ModelNet40 dataset [16] using the objects of the class person and manually segmenting the feet portion of each representation (Figure 5). To match the synthetic objects with the objects of the Miun-Feet dataset measured by the depth camera, we perform the following pre-processing tasks (Figure 3b):

- (1) Frontal projection: the depth camera does not capture the data the object itself obscures. Therefore, it is necessary to compute the frontal projection of each synthetic object with respect to the camera angle and remove the occluded data.
- (2) Dataset augmentation: The 3DHOG object descriptor is sensitive to the rotation. Thus, we perform dataset augmentation by rotating each synthetic object along the X-axis (0,15,30) degrees, the Y-axis (0,15,30) degrees and the Z-axis (0,30,60) degrees, giving a total of 27 rotations per synthetic object.

### 3.5. Point Cloud Object Segmentation

**Figure 6:**
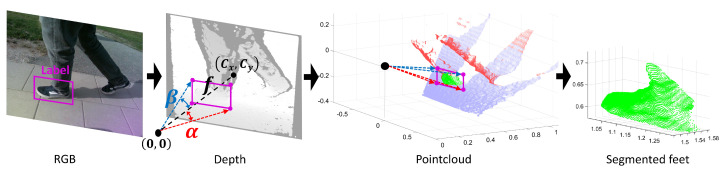
Point cloud object segmentation using the RGB labels.

The 3DHOG object descriptor requires point cloud data pre-processing to segment the caregiver’s feet, as shown in Figure 3b and Figure 6. The position of the feet is defined by the corresponding ground truth label file. We use a pinhole camera model and the depth camera intrinsic parameters to compute the angles α and β defined by the corners of the boundary boxes of each label with respect to the camera center as follows:

(1) $$\alpha =\arctan\frac{L_{c}-C_{x}}{f_{x}}\;\beta =\arctan\frac{L_{c}-C_{y}}{f_{y}},$$

where $L_{c}$ is the label corner in pixels, $C_{x}$ and $C_{y}$ are the camera center in pixels and $f_{x}$ and $f_{y}$ the camera focal length in pixels.

Label angles α and β for each label corner are then transferred to the point cloud representation. The caregiver’s feet are segmented by removing the data outside of the 3D rectangular pyramid defined by the label angles. Additionally, we perform a ground plane subtraction from the point cloud representation using the (M-estimator sample-consensus) MSAC algorithm [46].

### 3.6. YOLOv4-Tiny Approach

The YOLOv4-Tiny approach is evaluated in Experiment 1 (Figure 3a), and it is based on a compressed YOLOv4 approach [47] to make it less computationally and memory demanding. Therefore, it is particularly suitable for real-time operation in embedded systems. As a drawback, the target detection network of YOLOv4-Tiny is relatively simple, and therefore, the detection accuracy is lower than in YOLOv4, especially for smaller targets sizes [48]. However, in our application, the targets (feet) are relatively large with respect to the camera FoV.The YOLOv4-Tiny uses the CSPDarknet53 as the backbone network, which limits the input images to 1–3 channels. Hence, we modified the CSPDarknet53 to allow RGB-D (4 channels) input images. This is achieved by disabling the OPENCV option in the makefile and removing the limitation to 3 channels in the functions *hsv_rgb* and *rgb_hsv* from the file *image.c*. The used YOLOv4-Tiny hyperparameters are listed in Table 5.

### 3.7. 3DHOG Approach

The 3DHOG approach, evaluated in Experiment 2 (Figure 3b) uses a combination of the 3DHOG object descriptor and a Support Vector Machine (SVM) feature classifier. The 3DHOG [3] is a hand-crafted object descriptor based on the popular HOG [49]. Originally, the 3DHOG was developed for hazard detection in 3D scenes, and it has been extensively addressed in previous research [4,50]. As a drawback, the 3DHOG object descriptor leads to high-dimensional feature vector and therefore requires additional post-processing to reduce it and enable real-time performance. Hence, we computed the principal components analysis (PCA) of the 3DHOG feature matrix in order to select a reduced set of features that contain most of the initial data variance. The descriptor parameters and feature dimensionality for each voxel grid defined in Experiment 2 are summarized in Table 6 and Table 7, respectively.

### 3.8. Caregiver Distance and Angle Computation

**Figure 7:**
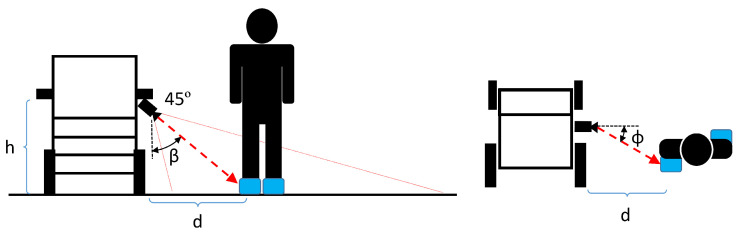
Caregiver distance and angle measurement.

We propose an alternative method to measure the relative distance and angle of the caregiver’s feet using only the RGB data. We used the center of each bounding box to compute the relative caregiver’s angle and distance. The ground truth data for the distance is measured by the depth channel. The ground truth for the angle is given by the annotated objects, and thus, the accuracy is correlated to the mAP. Distances are measured using the RGB data, assuming the GP is perpendicular to the PW along the detection range, and feet are located in the center of the predicted boundary boxes. The measurement setup is shown in Figure 7. Relative distances are computed using the intrinsic camera parameters as follows:

(2) $$\phi =\arctan\frac{C-C_{c}}{f}\;\alpha =\arctan\frac{R-R_{c}}{f}\;\beta =\left(\alpha -45\right)\;d=h\tan\beta ,$$

where *R* is the row coordinate of the predicted label, *C* is the column coordinate of the predicted label, $R_{c}$ is the camera height center in pixels, $C_{c}$ is the camera width center in pixels, α is the relative angle of the *R* with respect to the RGB camera, ϕ is the relative angle of the *C* with respect to the RGB camera, *f* is the focal length in pixels, β is the camera angle relative to the GP, *h* the camera height with respect to the GP in meters and *d* is the relative distance to the caregiver’s feet in meters.

## 4. Results and Analysis

### 4.1. Experiment 1. YOLOv4-Tiny

**Figure 8:**
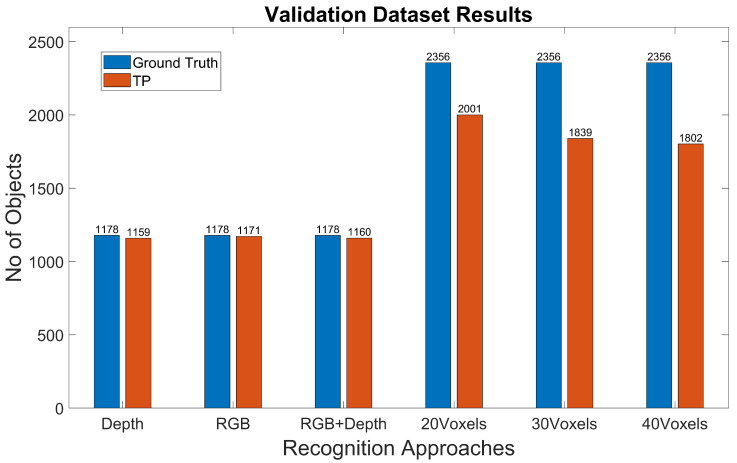
YOLOv4-Tiny results for the different test datasets using 1-channel depth, 3-channel RGB and 4-channel RGB+D input images.

**Figure 9:**
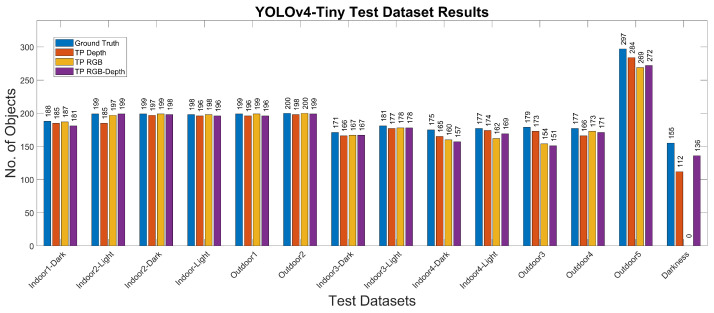
YOLOv4-Tiny results for the different test datasets using 1-channel depth, 3-channel RGB and 4-channel RGB+D input images.

**Figure 10:**
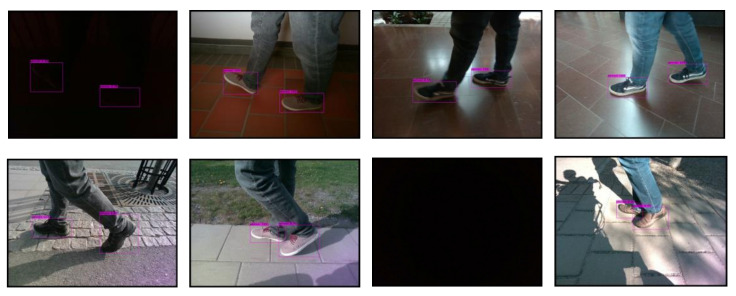
From top to bottom and left to right: YOLOv4-Tiny results using the 3-channel RGB frames for the 1-Indoor1-Dark, 2-Indoor1-Light, 3-Indoor2-Dark, 4-Indoor2-Light, 5-Outdoor1, 6-Outdoor2, 7-Darkness and 8-Outdoor5 scenarios.

The experimental results for the YOLOv4-Tiny approach for the different test cases and methods are summarized in Table 8 and Table 9 and Figure 8 and Figure 9. Figure 10 shows the detected bounding boxes. For all scenarios of the Miun-Feet validation dataset (Table 3), mAP is around 99% and thus has excellent performance. For the remaining Miun-Feet test datasets scenarios including different shoes and backgrounds, mAP drops to 96% and thus also has excellent performance. All analyzed methods do not show significant differences in terms of mAP. Only for the method RGB-D (4 channels), mAP is slightly lower (−2%) for the test scenarios. All test data were not considered in the training and validation steps. As expected for the Darkness scenario, the mAP drops to zero when using the RGB method. However, when we include the depth channel in the analysis, the mAP is around 95%, and therefore, it is possible to recognize the objects even in complete darkness. On the other hand, in low light conditions (Indoor1-Dark), it is possible to recognize the caregiver’s feet without decreasing the performance compared to the light and sunny scenarios.

### 4.2. Experiment 2: 3DHOG Approach

**Figure 11:**
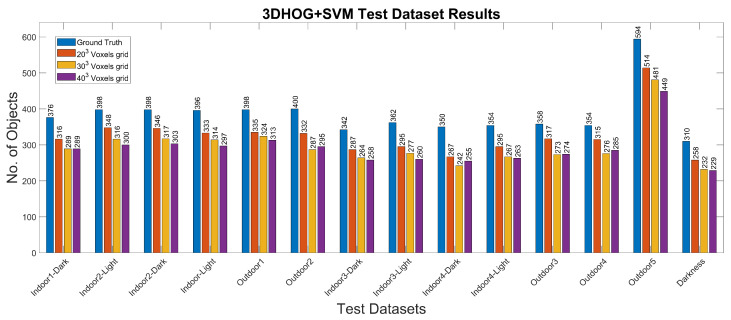
3DHOG results for the different test datasets using 1-channel depth, 3-channel RGB and 4-channel RGB+D input images.

**Figure 12:**
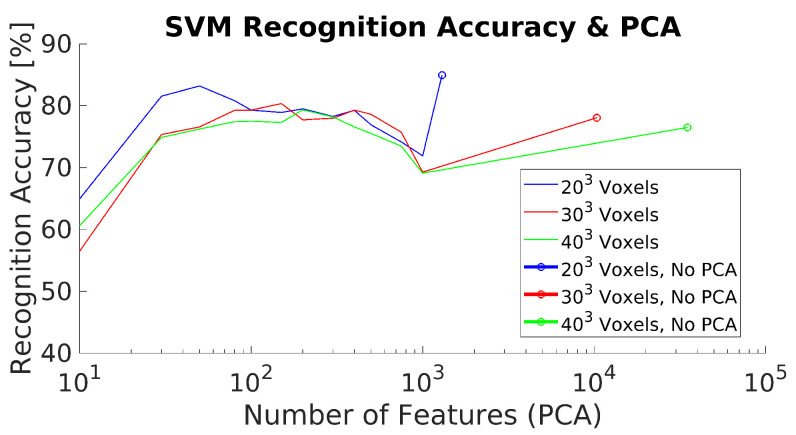
3DHOG mAP and PCA dimensionality feature reduction.

The experimental results for the 3DHOG approach for the different test cases and methods are summarized in Table 8 and Table 9 and Figure 11. The best mAP result is 84.93%, which is relatively lower than the results obtained with YOLOv4-Tiny in all analyzed test scenarios. Moreover, the 3DHOG results are better when the lower voxel grid ($20^{3}$ voxels) is used. Experiment 2 also evaluates the feature dimensionality reduction by applying PCA analysis Figure 12. The maximum number of Principal Components (PCs) are limited by the total number of synthetic objects (total_No) in the training dataset. Hence, it is not possible to reduce the initial number of features in more than Total_No-1 PC. The results show that feature dimensionality can be reduced without significant mAP loss. However, when we use a higher number of PCs (1000 PCs), the mAP drops significantly. These results match with the obtained result when using synthetic data for the test [4]. In addition, it is not possible to improve the mAP compared to the case where we use the full set of features.

### 4.3. Experiment 3: Caregiver’s Distance and Angle Computation

**Figure 13:**
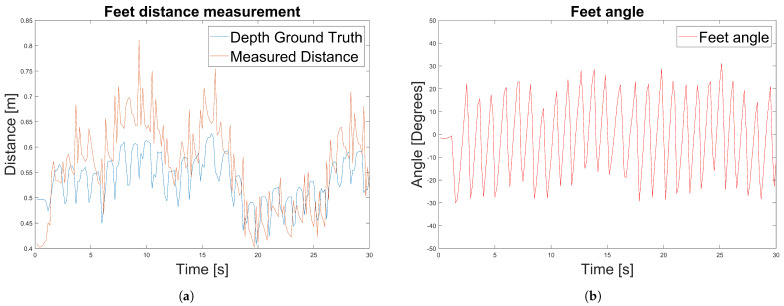
Experiment 3 results to measure the caregiver’s relative distance and angles using the RGB data. (**a**) Experiment 3: Caregiver’s distance measurement. (**b**) Experiment 3: Caregiver’s angle measurement.

Experimental results of a caregiver distance measurement are shown in Figure 13.

We used an outdoor test dataset for the experiment that contains 30 s of images of a caregiver walking beside the PW. We first measure the ground truth distances using the depth channel (Figure 13a). Measured distances using the depth channel are affected by the movement of the feet due to the walking motion and therefore are not constant over time. Then, we measured the distances and angles using the RGB data and the method introduced in Section 3.8. In the same way as for the feet distance measurements, the caregiver’s feet angle are affected by the feet motion but also by the motion of the PW.

**Figure 14:**
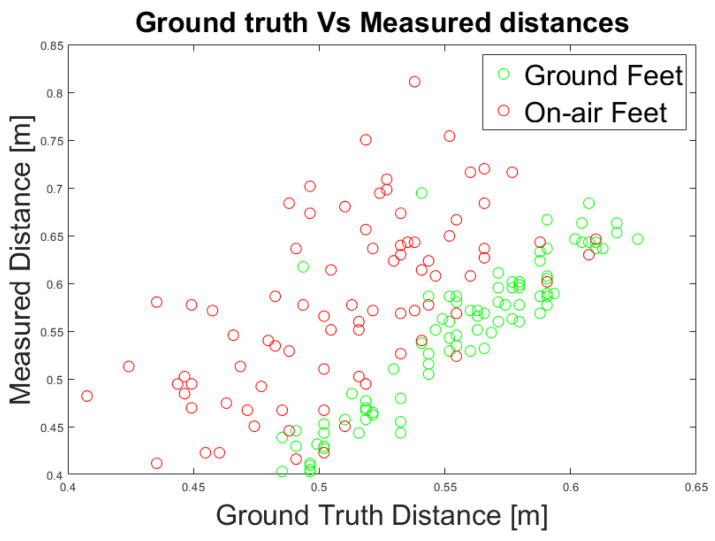
Experiment 3: Ground truth and measured distances using an RGB data.

Additionally, we evaluated the measured distances considering on-air feet not lying above the GP due to the walking motion (Figure 14). We consider that the on-air feet introduce an error in the distance measurement. Hence, we categorized each foot’s position for each frame according to its position above the GP. We evaluated the quality of the measurement with respect to the ground truth data by computing the linear regression of the whole feet data and only of the grounded feet, measuring the Root Mean Squared Error (RMSE) as a quality index. The evaluation shows that when the feet are above the GP, the distance measurements have a lower RMSE and, therefore, a higher degree of confidence (Table 10).

## 5. Discussion

### Evaluation of the Methods

Both recognition approaches analyzed perform with enough accuracy to recognize the objects. The YOLOv4-Tiny achieved good recognition results even when working in low lighting conditions, thus enabling the development of a reliable contactless driving system for PW and autonomous robotic applications. In addition, it is possible to recognize the objects in complete darkness using the depth channel (1-channel) or merging the RGB and depth channels (4-channels) without degrading the recognition performances, making it especially suitable for applications that require good and reliable object recognition performances despite the lighting conditions and light saturation artifacts.

The 3DHOG approach requires previous point cloud pre-processing to segment the objects from the unstructured 3D points. The pre-processing segmentation causes a higher computational cost but also leads to segmentation errors, which affect the recognition performance [5]. In Experiment 2, we used a new method of the object segmentation using the same labels we used for the YOLOv4-Tiny approach. As a result, object segmentation avoids errors, improves the recognition performances and allows us to compare both object recognition approaches regarding segmentation artifacts. However, the segmentation method only works on labeled data, and therefore, it is not valid for new unlabeled data frames. Hence, the proposed segmentation method does not solve the problem of having segmentation artifacts when using the 3DHOG and point cloud input data.

On the other hand, YOLOv4-Tiny requires, instead, a labeled dataset of images for training and validation, covering all the possible scene variations with respect to backgrounds, lighting, targets and obstacles. Hence, it is necessary to consider all the possible indoor and outdoor scenarios due to the high variability of environments where a PW is intended to be used, and as consequence, to collect and label a large dataset of images. This drawback can lead to recognition problems when the PW is used in a different scenario not considered during the training, although our experimental results are good even in this situation. In order to address this problem and to avoid the requirement of large labeled datasets, it is possible to use transfer learning [51] or few-shot [52] techniques.

YOLOv4-Tiny results show good recognition performances when using only the RGB data (3 channels). Therefore, it is not required to include the depth data for an object recognition application. This result enables using only a monocular RGB camera instead of a depth camera for a caregiver recognition application. As a drawback, a monocular RGB camera does not allow measuring relative distances of the caregiver’s feet and also does not enable caregiver detection in complete darkness conditions.

The experimental results for a caregiver distance measurement using only the RGB data results in an average error of 3 cm. However, we assumed that the GP is perpendicular to the PW throughout the caregiver’s detection range. In the case of not having a flat GP, this assumption can lead to distance miscalculations. This drawback especially affects outdoor scenarios when the GP is not flat. In order to solve this miscalculation caused by the feet motion and position above the GP, it is required to detect the feet position and measure its relative distance only when the feet are above the GP and thus discard the measurements when feet are on-air. Regarding the caregiver’s feet angle, it is not possible discard on-air or ground feet angle measurements due to the motion of the PW. Hence, it is required to perform an average of the measured caregiver’s feet angle to estimate the caregiver’s angle position according to the PW.

## 6. Conclusions

Despite the relatively good performances of the 3DHOG approach for 3D object recognition, the YOLOv4-Tiny approach outperforms it in all the test scenarios and light conditions evaluated on the Miun-Feet dataset. The experimental results show that including the depth channel does not improve the recognition accuracy compared to using only RGB channels. In addition, it is possible to compute the caregiver relative distances and angles using only the RGB data. Only in the case of complete darkness, the object recognition is improved using the depth channel. However, complete darkness is not a requirement for a PW operation. Therefore, we believe a monocular RGB camera is a better option for a contactless PW control application.

## Figures and Tables

**Table 1:** Intel RealSense D455 active depth camera configuration.

Configuration preset	High density
Frame Resolution	640 × 480 pixels
Aligned RGB and Depth streams	Yes
Frame Rate	6 fps
Exposure	156 ms Auto
Active lighting power	Maximum

**Table 2:** Miun-Feet dataset scenarios construction.

Scenario	Placement	Background	Light Conditions	Exposure	Shoes Model
Indoor1-Dark	Tunnel corridor	Tiles/wall	Low light	156 ms	1–5
Indoor1-Light	Tunnel corridor	Tiles/wall	LED lighting	156 ms	1–5
Indoor2-Dark	University corridor	Hall	Low light	156 ms	1–5
Indoor2-Light	University corridor	Hall	LED lighting	156 ms	1–5
Outdoor1	Pedestrian asphalt	Road	Sunny	Auto	1–5
Outdoor2	Pedestrian tiles	Grass	Sunny	Auto	1–5
Indoor3-Dark	Tunnel corridor	Tiles/wall	Low light	156 ms	6–8
Indoor3-Light	Tunnel corridor	Tiles/wall	LED lighting	156 ms	6–8
Indoor4-Dark	University corridor	Hall	Low light	156 ms	6–8
Indoor4-Light	University corridor	Hall	LED lighting	156 ms	6–8
Outdoor3	Pedestrian asphalt	Road	Sunny	Auto	6–8
Outdoor4	Pedestrian tiles	Grass	Sunny	Auto	6–8
Outdoor5	Pedestrian tiles	Bikes parking	Sunny	Auto	6–8
Darkness	Laboratory	Wall/chairs	Darkness	156 ms	6–8

**Table 3:** Miun-Feet training and validation datasets definition.

	Scenarios						
**Dataset**	**Indoor1-Dark** **(Feet/Empty)**	**Indoor1-Light** **(Feet/Empty)**	**Indoor2-Dark** **Feet/Empty**	**Indoor2-Light** **(Feet/Empty)**	**Outdoor1** **(Feet/Empty)**	**Outdoor2** **(Feet/Empty)**	**Total** **(Feet/Empty)**
**Training**	500/500	500/500	500/500	500/500	500/500	500/500	3000/3000
**Validation**	100/100	100/100	100/100	100/100	100/100	100/100	600/600

**Table 4:** Training, validation and test datasets definition for both YOLOv4-Tiny and 3DHOG recognition approaches.

	YOLOv4-Tiny				3DHOG			
	Dataset	No.Frames	No.Empty	No.Obj.	Dataset	No.Feet	No.Others	Total Obj.
**Training**	Real	3000	3000	5880	Synthetic	50 × 27	1350	2700
**Validation**	Real	600	600	1178	Real	1178	1178	2356
Test:								
Indoor1-Dark	Real	100	100	188	Real	188	188	376
Indoor1-Light	Real	100	100	199	Real	199	199	398
Indoor2-Dark	Real	100	100	199	Real	199	199	398
Indoor2-Light	Real	100	100	188	Real	188	188	376
Indoor3-Dark	Real	90	90	171	Real	171	171	342
Indoor3-Light	Real	90	90	181	Real	181	181	362
Indoor4-Dark	Real	90	90	175	Real	175	175	350
Indoor4-Light	Real	90	90	177	Real	177	177	354
Outdoor1	Real	100	100	199	Real	199	199	398
Outdoor2	Real	100	100	200	Real	199	200	400
Outdoor3	Real	90	90	179	Real	179	179	358
Outdoor4	Real	90	90	177	Real	177	177	354
Outdoor5	Real	150	150	297	Real	297	297	594
Darkness	Real	80	80	155	Real	155	155	310

**Table 5:** YOLOv4-Tiny hyperparameters.

Batch size	64
Sub-division	24
Width	640
Height	480
Channels	1(Depth), 3(RGB), 4(RGB-D)
Momentum	0.9
Decay	0.0005
Max.batch	2000
Burn in	500
Steps	1800, 1900
Scales	0.1, 0.1
Anchors	(10,14), (23,27) (37,58), (81,82) (135,169), (344,319)

**Table 6:** 3DHOG and feature dimensionality for a $20^{3}$, $30^{3}$ and $40^{3}$ voxel grids.

Voxel Grid	$20^{3}$	$30^{3}$	$40^{3}$
$N_{Blocks}$	1	8	27
$N_{Cells}$	8	8	8
$N_{Features}$	1296	10,368	34,992

**Table 7:** Descriptor configuration parameters.

Parameter	Value
$\varphi_{Bins}$	18
$\theta_{Bins}$	9
$Cell_{Size}$	6
$Block_{Size}$	2
$Step_{Size}$	2

**Table 8:** mAP(%) results for the different test datasets and recognition approaches using the same shoes and scenarios used in the training dataset.

Method	Test Cases						
	**Validation** **mAP(%)**	**Indoor1-Dark** **mAP(%)**	**Indoor1-Light** **mAP(%)**	**Indoor2-Dark** **mAP(%)**	**Indoor2-Light** **mAP(%)**	**Outdoor1** **mAP(%)**	**Outdoor2** **mAP(%)**
Depth (1CH)	99.24	99.33	100	99.46	99.88	99.91	99.19
RGB (3CH)	99.83	99.98	100	100	99.78	100	100
RGB-D (4CH)	99.52	99.82	100	99.96	99.97	99.83	99.85
3DHOG, $20^{3}$ Voxels	84.93	84.04	87.44	86.93	84.09	84.17	83
3DHOG, $30^{3}$ Voxels	78.06	76.86	79.40	79.65	79.29	81.41	71.75
3DHOG, $40^{3}$ Voxels	76.49	76.86	75.38	76.13	75.00	78.64	73.75

**Table 9:** mAP(%) results for the different test datasets and recognition approaches using new shoes and including additional scenarios.

Method	Additional Test Cases							
	**Indoor3-Dark** **mAP(%)**	**Indoor3-Light** **mAP(%)**	**Indoor4-Dark** **mAP(%)**	**Indoor4-Light** **mAP(%)**	**Outdoor3** **mAP(%)**	**Outdoor4** **mAP(%)**	**Outdoor5** **mAP(%)**	**Darkness** **mAP(%)**
Depth (1CH)	98.66	99.39	96.45	98.78	99.10	97.61	99.17	97.68
RGB (3CH)	99.33	99.99	97.63	98.50	96.15	99.32	96.70	0
RGB-D (4CH)	98.83	99.88	95.46	97.58	98.64	95.92	96.23	94.98
3DHOG, $20^{3}$ Voxels	83.92	81.49	76.29	83.33	88.55	88.98	86.53	83.23
3DHOG, $30^{3}$ Voxels	77.19	76.52	69.14	75.42	76.26	77.97	80.98	74.84
3DHOG, $40^{3}$ Voxels	75.44	71.82	72.86	74.29	76.54	80.51	75.59	73.87

**Table 10:** Root mean squared error of caregiver distance measurement.

	All Feet	Ground Feet
RMSE (m)	0.077	0.035

## Data Availability

Miun-Feet dataset used in this work is available at https://doi.org/10.5878/k44d-3y06, (accessed on 29 November 2021).
